# Evaluating the capability of large language models in radiotherapy through professional certification examinations in Japan

**DOI:** 10.1093/jrr/rraf083

**Published:** 2026-01-10

**Authors:** Noriyuki Kadoya, Yoshiyuki Takahashi, Seiya Koga, Hikaru Tanno, Kazuhiro Arai, Shohei Tanaka, Yoshiyuki Katsuta, Hinako Harada, So Omata, Takaya Yamamoto, Rei Umezawa, Ken Takeda, Keiichi Jingu

**Affiliations:** Department of Radiation Oncology, Tohoku University Graduate School of Medicine, 1-1 Seiryo-machi, Aoba-ku, Sendai, Miyagi 980-8574, Japan; Department of Radiation Oncology, Tohoku University Graduate School of Medicine, 1-1 Seiryo-machi, Aoba-ku, Sendai, Miyagi 980-8574, Japan; Department of Radiation Oncology, Tohoku University Graduate School of Medicine, 1-1 Seiryo-machi, Aoba-ku, Sendai, Miyagi 980-8574, Japan; Department of Radiation Oncology, Tohoku University Graduate School of Medicine, 1-1 Seiryo-machi, Aoba-ku, Sendai, Miyagi 980-8574, Japan; Department of Radiation Oncology, Tohoku University Graduate School of Medicine, 1-1 Seiryo-machi, Aoba-ku, Sendai, Miyagi 980-8574, Japan; Department of Radiation Oncology, Tohoku University Graduate School of Medicine, 1-1 Seiryo-machi, Aoba-ku, Sendai, Miyagi 980-8574, Japan; Department of Radiation Oncology, Tohoku University Graduate School of Medicine, 1-1 Seiryo-machi, Aoba-ku, Sendai, Miyagi 980-8574, Japan; Department of Radiation Oncology, Tohoku University Graduate School of Medicine, 1-1 Seiryo-machi, Aoba-ku, Sendai, Miyagi 980-8574, Japan; Department of Radiation Oncology, Tohoku University Graduate School of Medicine, 1-1 Seiryo-machi, Aoba-ku, Sendai, Miyagi 980-8574, Japan; Department of Radiation Oncology, Tohoku University Graduate School of Medicine, 1-1 Seiryo-machi, Aoba-ku, Sendai, Miyagi 980-8574, Japan; Department of Radiation Oncology, Tohoku University Graduate School of Medicine, 1-1 Seiryo-machi, Aoba-ku, Sendai, Miyagi 980-8574, Japan; Course of Radiological Technology, Health Sciences, Tohoku University Graduate School of Medicine, 2-1 Seiryo-machi, Aoba-ku, Sendai, 980-8575, Japan; Department of Radiation Oncology, Tohoku University Graduate School of Medicine, 1-1 Seiryo-machi, Aoba-ku, Sendai, Miyagi 980-8574, Japan

**Keywords:** radiotherapy, LLM, artificial intelligence, medical physicist, radiation oncologist

## Abstract

Large language models (LLMs), such as ChatGPT and Grok, have rapidly advanced in natural language understanding and are increasingly being applied to specialized fields, including medicine. In this study, we evaluated the domain-specific knowledge of LLMs in radiotherapy by assessing their performance on three certification examinations in Japan: the Japanese Medical Physicist Examination, the Japanese Board Examination for Radiologists and the Japanese Board Examination for Radiation Oncologists. We assessed five LLMs—ChatGPT-5, ChatGPT-5 Pro, Grok 4, Grok 4 heavy and Gemini 2.5 Pro—by inputting all multiple-choice questions from these exams into each model and recording their responses. The AI-generated answers were compared with reference answers determined by experienced medical physicists and radiation oncologists. The results demonstrated average accuracies of 84.7 ± 2.0% (ChatGPT-5), 94.7 ± 2.1% (ChatGPT-5 Pro), 78.4 ± 1.2% (Grok 4), 81.6 ± 2.2% (Grok 4 heavy) and 88.9 ± 1.2% (Gemini 2.5 Pro). All models achieved over 75% accuracy, with ChatGPT-5 Pro consistently outperforming others, attaining an average accuracy exceeding 90% across all examinations. These findings highlight the strong potential of advanced LLMs, particularly ChatGPT-5 Pro, for future integration into radiotherapy-related applications such as automated contouring and treatment planning support.

## INTRODUCTION

Large language models (LLMs), such as ChatGPT and Grok, have rapidly advanced in natural language understanding and generation through training on extensive corpora comprising articles, books and internet-based content [1]. Recent developments as of July 2025—such as OpenAI’s GPT-4o Pro, xAI’s Grok 4 and Google DeepMind’s Gemini 2.5 Pro—have further extended the capabilities of these models in specialized domains [2–4]. In the medical field, Gilson *et al*. evaluated ChatGPT’s performance on the United States Medical Licensing Examination, with accuracy rates ranging from 42 to 64.4% across four datasets [5]. Med-PaLM 2, a medically fine-tuned variant of Google’s PaLM 2, demonstrated near-expert performance with an 86.5% accuracy rate [6].

The application of LLMs in radiation-related disciplines has also gained attention. Rebelo *et al*. developed an AI-assisted chatbot to facilitate knowledge transfer about radiotherapy for patients, families and the public, demonstrating the chatbot’s effectiveness in delivering accurate information [7]. Liu *et al*. introduced Radiology-GPT, a radiology-specific transformer model that outperformed existing baselines in multiple evaluations [8]. In radiation oncology, Dennstadt *et al*. reported a 60.61% answer accuracy by ChatGPT on field-specific content [9]. More recently, Wang *et al*. showed that newly released LLMs achieved expert-level performance on radiation oncology physics questions [10]. While such studies highlight the growing potential of LLMs in medical applications, their capabilities in non-English and localized settings remain underexplored. In particular, the performance of state-of-the-art LLMs—such as ChatGPT, Grok and Gemini—on Japanese-language medical examinations in the field of radiotherapy has not yet been systematically assessed.

In this study, we aimed to evaluate the domain-specific knowledge of modern LLMs in radiotherapy by testing their performance on three certification examinations in Japan: the Japanese Medical Physicist Examination, the Japanese Board Examination for Radiologists and the Japanese Board Examination for Radiation Oncologists. This benchmarking effort serves as a foundation for understanding the readiness and limitations of LLMs in supporting medical professionals in Japanese-language clinical and educational settings.

## MATERIALS AND METHODS

### L‌LM models

In this study, we evaluated five LLMs: ChatGPT-5 (OpenAI, CA, USA), ChatGPT-5 Pro (OpenAI), Grok 4 (xAI, CA, USA), Grok 4 Heavy and Gemini 2.5 Pro (Google, CA, USA). ChatGPT, developed by OpenAI and initially released in 2022, is capable of generating fluent, human-like text in multiple languages, including English, Spanish, French and Japanese. The most recent version, ChatGPT-5, became available in August 2025. Grok, developed by xAI, was first released in 2024, with the latest iteration—Grok 4—launched in July 2025. Gemini, developed by Google and introduced in 2023, also saw its most recent release (Gemini 2.5 Pro) in June 2025.

### Evaluation

We utilized questions from three radiotherapy-related professional board examinations administered in Japan: the Japanese Medical Physicist Board Examination, the Japanese Board Examination for Radiologists and the Japanese Board Examination for Radiation Oncologists from the years 2020 to 2023. These examinations encompass a variety of question formats, including multiple-choice questions. In the Japanese medical system, medical physicists specializing in radiation therapy are required to pass the Medical Physicist Board Examination. Radiation oncologists typically begin their certification pathway by passing the Radiologist Board Examination, followed by the specialized Radiation Oncologist Board Examination. Therefore, we considered that evaluating performance across these three examinations offers a comprehensive assessment of domain-specific knowledge in radiation therapy as it pertains to two key professional groups in Japan.

For this study, we focused exclusively on multiple-choice questions to assess the text-based reasoning capabilities of LLMs. Questions involving visual materials (e.g. radiographic images), questions deemed inappropriate or ambiguous by expert reviewers, and those whose correct answers had changed due to updates in clinical guidelines between the time of exam administration and the present were excluded. Questions were excluded for three reasons: (i) conflicts with updated guidelines, (ii) inclusion of image-based items, and (iii) items judged inappropriate by the answer authors. The numbers of excluded items were as follows: in the 2020 examinations, 5, 3 and 4; in 2021, 13, 0 and 8; in 2022, 12, 9 and 12 and in 2023, 14, 1 and 10, from the medical physicist, radiologist, and radiation oncologist examinations, respectively.

All questions were first input into each LLM in a single batch. In cases where the model failed to generate complete outputs or prematurely terminated the response (e.g. producing partial explanations or halting mid-answer), each question was re-entered individually to ensure that valid responses were obtained for all items. Finally, we compared AI-based answers with the correct answers that were determined by experienced medical physicists and radiation oncologists, since the correct answers are not provided by these Japanese Board examinations. All questions were entered into all LLMs from July to October 2025.

## RESULTS

### Medical physicist examination

The average accuracy rates for the Japanese Medical Physicist Board Examination were as follows: ChatGPT-5, 88.0 ± 2.3%; ChatGPT-5 Pro, 95.9 ± 1.8%; Grok 4, 83.7 ± 4.3%; Grok 4 Heavy, 88.5 ± 3.1% and Gemini 2.5 Pro, 91.4 ± 2.3%. The maximum difference in accuracy among the five LLMs was 12.2%, with ChatGPT-5 Pro achieving the highest performance.

### Radiologist examination

For the Japanese Board Examination for Radiologists, the average accuracy rates were: ChatGPT-5, 86.1 ± 1.1%; ChatGPT-5 Pro, 95.6 ± 2.3%; Grok 4, 79.9 ± 4.1%; Grok 4 Heavy, 78.7 ± 2.5% and Gemini 2.5 Pro, 91.5 ± 1.9%. The performance gap reached 16.9%, with ChatGPT-5 Pro again demonstrating the highest accuracy.

### Radiation oncologist examination

In the Japanese Board Examination for Radiation Oncologists, the average accuracy rates were: ChatGPT-5, 75.4 ± 6.2%; ChatGPT-5 Pro, 90.3 ± 3.8%; Grok 4, 63.9 ± 4.9%; Grok 4 Heavy, 70.4 ± 7.3% and Gemini 2.5 Pro, 79.0 ± 2.2%. The difference between the highest and lowest performing models was 26.5%, with ChatGPT-5 Pro once again achieving the top score.

### Overall accuracy

**Fig. 1:**
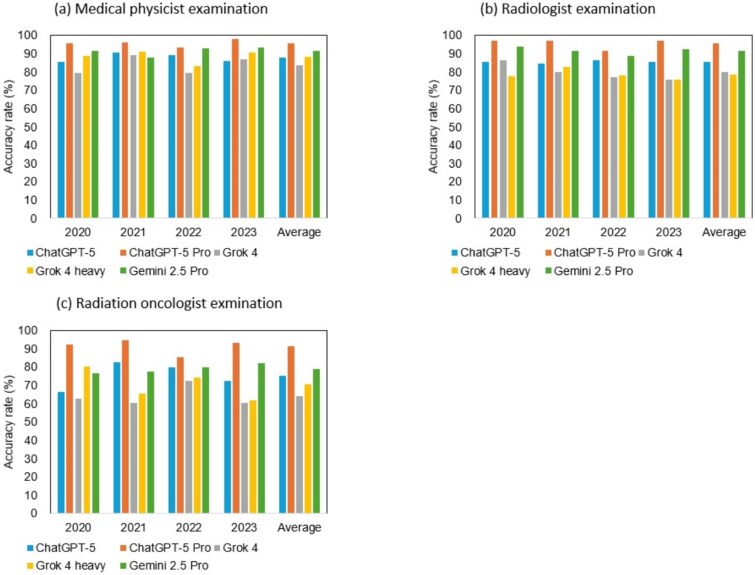
Summary of the overall accuracy rate of five LLMs.

Table 1 and Fig. 1 summarize the accuracy rates across all examinations. The overall mean accuracy rates for each LLM across the three examinations were: ChatGPT-5, 84.7 ± 2.0%; ChatGPT-5 Pro, 94.7 ± 2.1%; Grok 4, 78.4 ± 1.2%; Grok 4 Heavy, 81.6 ± 2.2% and Gemini 2.5 Pro, 88.9 ± 1.2%. All models demonstrated an average accuracy exceeding 70%, with ChatGPT-5 Pro approaching 95%, indicating near-expert level performance in this domain.

**Table 1:** Summary of accuracy rate for each category

	Accuracy rate (%)						Accuracy rate (%)						Accuracy rate (%)						Accuracy rate (%)						Accuracy rate (%)				
	2020						2021						2022						2023						Average				
category	Number of questions	ChatGPT-5	ChatGPT-5 Pro	Grok 4	Grok 4 heavy	Gemini 2.5 Pro	Number of questions	ChatGPT-5	ChatGPT-5 Pro	Grok 4	Grok 4 heavy	Gemini 2.5 Pro	Number of questions	ChatGPT-5	ChatGPT-5 Pro	Grok 4	Grok 4 heavy	Gemini 2.5 Pro	Number of questions	ChatGPT-5	ChatGPT-5 Pro	Grok 4	Grok 4 heavy	Gemini 2.5 Pro	ChatGPT-5	ChatGPT-5 Pro	Grok 4	Grok 4 heavy	Gemini 2.5 Pro
**Medical physicist examination**																													
basic medicine	20	90.0	95.0	85.0	90.0	95.0	17	100.0	100.0	94.1	100.0	100.0	18	94.4	88.9	83.3	83.3	94.4	18	88.9	94.4	94.4	94.4	94.4	93.3 ± 4.4	94.6 ± 3.9	89.2 ± 5.1	91.9 ± 6.1	96.0 ± 2.4
diagnostic radiology	10	90.0	100.0	100.0	100.0	90.0	9	88.9	100.0	100.0	100.0	88.9	9	88.9	88.9	88.9	88.9	88.9	9	88.9	100.0	88.9	100.0	100.0	89.2 ± 0.5	97.2 ± 4.8	94.4 ± 5.6	97.2 ± 4.8	91.9 ± 4.7
nuclear medicine	10	100.0	100.0	80.0	90.0	100.0	10	80.0	90.0	90.0	90.0	80.0	10	90.0	100.0	90.0	90.0	90.0	10	90.0	100.0	90.0	100.0	100.0	90.0 ± 7.1	97.5 ± 4.3	87.5 ± 4.3	92.5 ± 4.3	92.5 ± 8.3
radiation oncology	10	100.0	100.0	90.0	90.0	100.0	10	90.0	100.0	100.0	90.0	100.0	10	80.0	100.0	80.0	80.0	100.0	10	100.0	100.0	100.0	100.0	90.0	92.5 ± 8.3	100.0 ± 0.0	92.5 ± 8.3	90.0 ± 7.1	97.5 ± 4.3
radiation biology	10	80.0	100.0	90.0	90.0	90.0	9	88.9	100.0	88.9	88.9	100.0	9	100.0	100.0	100.0	100.0	100.0	10	100.0	90.0	100.0	100.0	100.0	92.2 ± 8.4	97.5 ± 4.3	94.7 ± 5.3	94.7 ± 5.3	97.5 ± 4.3
radiation physics	15	80.0	93.3	86.7	86.7	93.3	15	100.0	100.0	86.7	86.7	100.0	14	100.0	92.9	85.7	100.0	92.9	14	100.0	100.0	100.0	100.0	100.0	95.0 ± 8.7	96.5 ± 3.5	89.8 ± 5.9	93.3 ± 6.7	96.5 ± 3.5
statistics	5	80.0	100.0	60.0	100.0	100.0	4	100.0	100.0	100.0	100.0	100.0	4	100.0	100.0	100.0	100.0	100.0	4	100.0	100.0	100.0	100.0	100.0	95.0 ± 8.7	100.0 ± 0.0	90.0 ± 17.3	100.0 ± 0.0	100.0 ± 0.0
health physics/radiological protection	10	80.0	100.0	70.0	100.0	100.0	9	100.0	100.0	66.7	100.0	66.7	10	90.0	80.0	60.0	70.0	80.0	10	40.0	100.0	90.0	100.0	100.0	77.5 ± 22.8	95.0 ± 8.7	71.7 ± 11.2	92.5 ± 13.0	86.7 ± 14.1
diagnostic radiation physics	10	80.0	100.0	70.0	80.0	70.0	10	80.0	90.0	70.0	70.0	70.0	10	100.0	100.0	70.0	90.0	100.0	8	87.5	100.0	100.0	100.0	100.0	86.9 ± 8.2	97.5 ± 4.3	77.5 ± 13.0	85.0 ± 11.2	85.0 ± 15.0
nuclear medicine physics	10	70.0	70.0	50.0	60.0	70.0	8	87.5	100.0	100.0	100.0	100.0	10	70.0	90.0	80.0	50.0	80.0	9	77.8	88.9	66.7	77.8	88.9	76.3 ± 7.2	87.2 ± 10.8	74.2 ± 18.3	71.9 ± 19.0	84.7 ± 11.1
radiation therapy physics	8	100.0	100.0	87.5	100.0	100.0	8	100.0	100.0	100.0	87.5	100.0	7	85.7	85.7	71.4	85.7	85.7	6	83.3	100.0	83.3	83.3	66.7	92.3 ± 7.8	96.4 ± 6.2	85.6 ± 10.2	89.1 ± 6.4	88.1 ± 13.7
radiation metrology	8	100.0	100.0	75.0	100.0	100.0	9	88.9	88.9	88.9	88.9	77.8	10	80.0	90.0	50.0	50.0	90.0	9	100.0	100.0	66.7	77.8	88.9	92.2 ± 8.4	94.7 ± 5.3	70.1 ± 14.1	79.2 ± 18.6	89.2 ± 7.9
medical and imaging informatics	9	88.9	88.9	88.9	88.9	88.9	9	88.9	88.9	100.0	100.0	100.0	7	100.0	100.0	85.7	100.0	100.0	9	100.0	100.0	100.0	100.0	100.0	94.4 ± 5.6	94.4 ± 5.6	93.6 ± 6.4	97.2 ± 4.8	97.2 ± 4.8
radiation-related laws and recommendations/medical ethics	10	60.0	100.0	60.0	80.0	90.0	10	80.0	90.0	70.0	80.0	40.0	10	70.0	100.0	80.0	90.0	100.0	10	50.0	100.0	30.0	30.0	70.0	65.0 ± 11.2	97.5 ± 4.3	60.0 ± 18.7	70.0 ± 23.5	75.0 ± 22.9
Total (all categories)	145	85.5	95.9	79.3	89.0	91.7	137	91.2	96.4	89.1	91.2	87.6	138	89.1	93.5	79.7	83.3	92.8	136	86.0	97.8	86.8	90.4	93.4	88.0 ± 2.3	95.9 ± 1.6	83.7 ± 4.3	88.5 ± 3.1	91.4 ± 2.3
**Radiologist examination**																													
diagnostic radiology	41	80.5	92.7	87.8	70.7	87.8	52	84.6	96.2	75.0	76.9	88.5	49	83.7	89.8	75.5	75.5	83.7	51	80.4	96.1	68.6	68.6	90.2	82.3 ± 1.9	93.7 ± 2.6	76.7 ± 6.9	72.9 ± 3.4	87.5 ± 2.4
interventional radiology	2	50.0	100.0	100.0	100.0	100.0	3	100.0	100.0	66.7	100.0	100.0	2	100.0	100.0	100.0	100.0	100.0	3	33.3	66.7	33.3	33.3	100.0	70.8 ± 29.8	91.7 ± 14.4	75.0 ± 27.6	83.3 ± 28.9	100.0 ± 0.0
nuclear medicine	10	90.0	100.0	90.0	100.0	100.0	15	93.3	100.0	93.3	93.3	93.3	15	93.3	93.3	86.7	86.7	93.3	15	93.3	100.0	93.3	93.3	100.0	92.5 ± 1.4	98.3 ± 2.9	90.8 ± 2.8	93.3 ± 4.7	96.7 ± 3.3
radiation oncology	17	100.0	100.0	82.4	76.5	100.0	22	77.3	95.5	86.4	86.4	90.9	22	90.9	95.5	77.3	72.7	95.5	22	90.9	100.0	81.8	81.8	90.9	89.8 ± 8.1	97.7 ± 2.3	81.9 ± 3.2	79.3 ± 5.2	94.3 ± 3.8
general radiological knowledge	11	100.0	100.0	81.8	81.8	100.0	13	84.6	100.0	76.9	84.6	100.0	8	75.0	87.5	62.5	87.5	87.5	13	100.0	100.0	84.6	84.6	92.3	89.9 ± 10.7	96.9 ± 5.4	76.5 ± 8.5	84.6 ± 2.0	95.0 ± 5.3
Total (all categories)	81	87.7	96.3	86.4	77.8	93.8	105	84.8	97.1	80.0	82.9	91.4	96	86.5	91.7	77.1	78.1	88.5	104	85.6	97.1	76.0	76.0	92.3	86.1 ± 1.1	95.6 ± 2.3	79.9 ± 4.1	78.7 ± 2.5	91.5 ± 1.9
**Radiation oncologist exmination**																													
Total (all categories)	51	66.7	88.2	62.7	80.4	76.5	58	82.8	94.8	60.3	65.5	77.6	54	79.6	85.2	72.2	74.1	79.6	73	72.6	93.2	60.3	61.6	82.2	75.4 ± 6.2	90.3 ± 3.8	63.9 ± 4.9	70.4 ± 7.3	79.0 ± 2.2
**All exminations**	277	82.7	94.6	78.3	84.1	89.2	300	87.0	96.3	80.3	83.3	87.0	288	86.5	91.3	78.1	79.9	88.9	313	82.7	96.5	77.0	78.9	90.4	84.7 ± 2.0	94.7 ± 2.1	78.4 ± 1.2	81.6 ± 2.2	88.9 ± 1.2

### Error pattern analysis

**Fig. 2:**
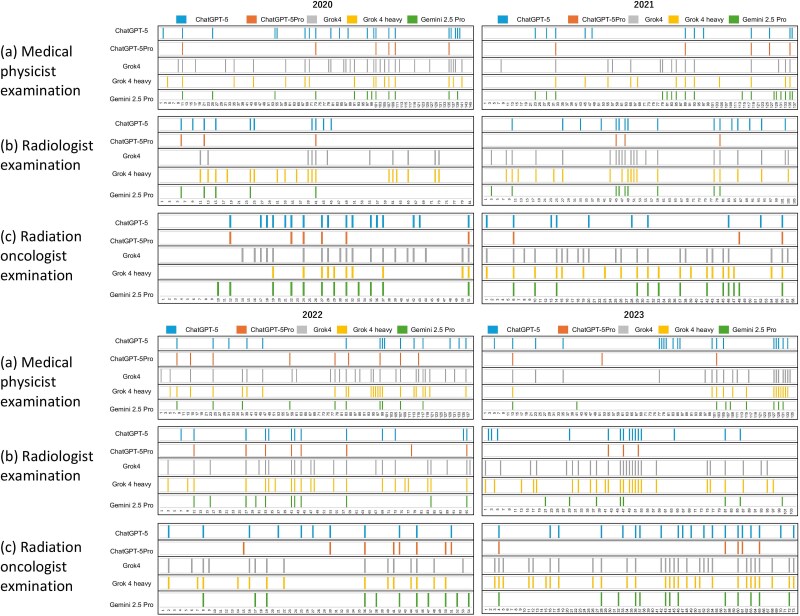
Distribution of incorrectly selected answer by each LLM across six exam sets. Each row shows one LLM. Numbers on the *x*-axis indicate question IDs, and colored bars represent incorrect answers.

**Fig. 3:**
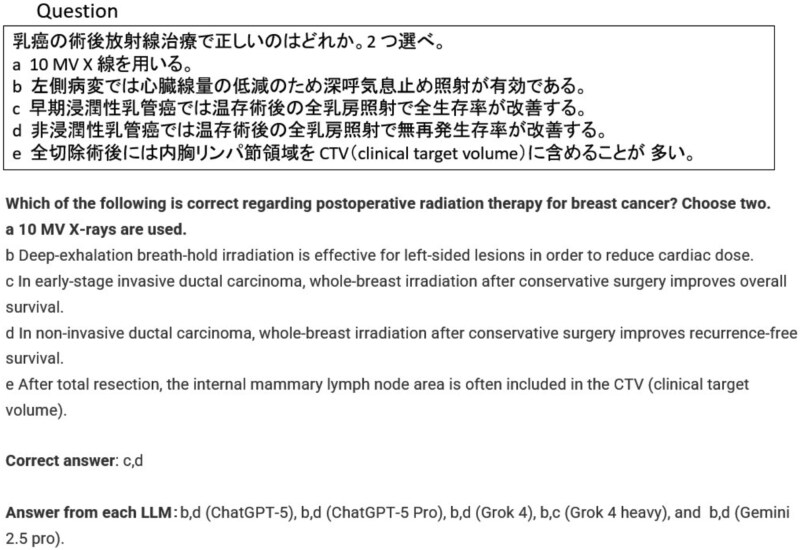
Example question in the case that all LLMs are not correct (question 80 in radiologist examination 2021).

**Fig. 4:**
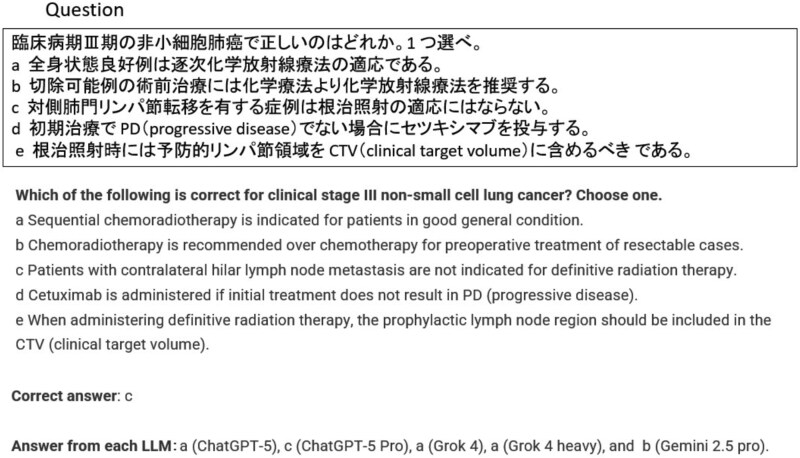
Example question in the case where only ChatGPT-5 Pro answered correctly (question 78 in radiologist examination 2021).

Figure 2 illustrates the distribution of incorrectly selected answers by each LLM across the six examination datasets. To provide a qualitative perspective, two representative cases are highlighted: one in which all five LLMs answered incorrectly (Fig. 3), and another in which only ChatGPT-5 Pro provided the correct response (Fig. 4).

## DISCUSSION

In this study, we evaluated the performance of five LLMs in answering questions from three Japanese board certification examinations related to radiotherapy: the Medical Physicist, Radiologist, and Radiation Oncologist examinations. Notably, ChatGPT-5 Pro achieved average accuracy rates exceeding 90% across all three examinations, demonstrating substantial potential for application in the field of radiation medicine.

Our findings are consistent with those of Wang *et al*., who assessed the performance of five LLMs—ChatGPT-o1-preview, GPT-4o, LLaMA 3.1, Gemini 1.5 Pro and Claude 3.5 Sonnet—on radiation oncology physics questions [10]. In their study, ChatGPT-o1-preview, the predecessor of ChatGPT-o3-pro, demonstrated the highest performance. The alignment of their results with ours suggests that LLMs maintain high levels of domain understanding when responding to inputs in both English and Japanese. Importantly, this study is the first to demonstrate such performance not only in the Japanese Medical Physicist Examination but also in the Radiologist and Radiation Oncologist examinations, reinforcing the feasibility of utilizing LLMs in Japanese-language radiotherapy-related applications [11].

In the example shown in Fig. 3, all LLMs, including ChatGPT-5 Pro, incorrectly selected the statement ‘deep expiration breath-hold irradiation is effective for reducing cardiac dose in left-sided breast cancer’. The correct statement should instead refer to ‘deep inspiration breath-hold irradiation’. This suggests that the models may have confused ‘呼気 (exhalation)’ with ‘
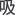
気 (inhalation)’. The likely reason for this misinterpretation is that the two characters differ by only one Chinese character (‘呼’ vs. ‘
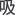
’), which are semantically opposite but visually and morphologically similar. When Japanese text was converted into vector representations during tokenization, these characters might occupy nearby positions in the embedding space, leading to the incorrect association at the semantic-vector level. Conversely, Fig. 4 presents an instance in which only ChatGPT-5 Pro correctly answered a question regarding radiotherapy strategies for stage III lung cancer. Correctly identifying option B as inappropriate required awareness that chemotherapy alone is not generally recommended for stage III disease unless radiation is contraindicated. Furthermore, understanding that concurrent chemoradiotherapy is superior to sequential therapy in patients with good performance status was also necessary. These results suggest that ChatGPT-5 Pro has a robust grasp of nuanced clinical guidelines, extending beyond surface-level knowledge. In general, chatGPT’s tendency to outperform other LLMs is likely due to its superior capabilities in (i) its ability to keep up with the latest information and search linkage accuracy, (ii) its accuracy in generating long sentences and understanding complex support, and (iii) its high level of logical thinking and execution ability. Similar trends were observed in Table 1. ChatGPT-5 Pro, which achieved the best overall ranking, had the lowest accuracy in the 2020 medical physicist exam category: nuclear medicine physics. Incorrect answers tended to include context-dependent abbreviations such as PET and SPECT mixed in with Japanese, and this may be an area where chatGPT 5 Pro was weak.

Numerous studies have explored LLM-driven applications in radiotherapy. Wang *et al*. proposed GPT-Plan, a multi-agent system based on the GPT-4 architecture for automated, iterative optimization of radiotherapy plans [12]. Oh *et al*. developed a LLM–driven system for multimodal target volume contouring [13], while Dong *et al*. proposed a method for LLM-based prediction of 3D dose distribution in treatment planning [14]. These studies collectively underscore the expanding role of LLMs in radiotherapy workflows, particularly in planning and contouring. For such applications to be clinically viable, however, it is critical that LLMs possess reliable and comprehensive domain knowledge. Our findings provide valuable evidence that such domain knowledge can be accessed through Japanese-language inputs, an area that has been underrepresented in prior research. It is also worth noting that all models used in this study were cloud-based. While effective, future clinical integration may necessitate the use of local deployment options, particularly for embedding LLMs into radiotherapy planning systems or electronic health records. Addressing data privacy, regulatory and infrastructural challenges associated with such integration will be crucial.

This study has several limitations. First, we did not apply advanced prompt engineering techniques, which have been shown in prior work to significantly influence LLM output and task performance. Prompt engineering, which involves optimizing the input instructions provided to the LLM, can affect model performance by guiding it toward more accurate and contextually appropriate outputs [15, 16]. Second, we did not use repeated prompts for each question. Previous studies have reported that most modern LLMs demonstrate relatively low sensitivity to prompt repetition [17]. Although reproducibility was not the main focus, we conducted preliminary repeat tests. ChatGPT-5 showed slight accuracy changes (89.2% → 89.2% in 2022; 84.6% → 85.6% in 2023 with radiologist exam), and Gemini 2.5 Pro showed 76.5% → 78.4% on the 2020 radiation oncologist exam. Some variation was observed, but these differences did not materially affect the study’s overall findings.

## CONCLUSIONS

We evaluated the performance of modern five LLMs in answering the three radiotherapy-related medical staff board examination questions, setting a benchmark for this LLM. Among the models tested, ChatGPT-5 Pro consistently demonstrated the highest accuracy across all examinations, achieving an average accuracy rate exceeding 90%. These findings highlight the significant potential of modern LLMs—particularly ChatGPT-5 Pro—for integration into radiotherapy applications, including automated contouring, treatment planning and clinical decision support systems.
